# Hemopexin and Cancer

**DOI:** 10.3390/ijms23020997

**Published:** 2022-01-17

**Authors:** Veronica Fiorito, Emanuela Tolosano

**Affiliations:** Molecular Biotechnology Center, Department of Molecular Biotechnology and Health Sciences, University of Torino, 10126 Torino, Italy; emanuela.tolosano@unito.it

**Keywords:** hemopexin, cancer, heme

## Abstract

Hemopexin is the plasma protein with the highest affinity for heme. Seminal studies have highlighted its role in different kinds of heme-associated disorders, but its implication in cancer has been neglected for a long time. Considering the emerging importance of heme in tumors, the present review proposes an update of the works investigating hemopexin involvement in cancer, with the attempt to stimulate further future studies on this topic.

## 1. Introduction

Hemopexin (Hx) is a 60-kDa plasma glycoprotein mainly expressed by the liver, although the nervous system, skeletal muscle, retina, and kidney are also recognized Hx synthesis sites. Under physiological conditions, the concentration of Hx in plasma is 0.5–1 mg/mL, but during inflammatory events its level increases due to interleukin (IL)-6, IL-11, IL-1b, leukemia inhibitory factor, oncostatin M, and tumor necrosis factor (TNF)-a stimulation [1], Hx being an acute-phase protein [2]. The first studies on Hx date back to about 40 years ago [1]. Since then, Hx has been considered the plasma protein with the highest affinity for heme and the ultimate scavenger of labile heme.

A wide range of events can account for the presence of heme in plasma. During erythrophagocytosis a large amount of heme is released in the macrophage, and although it is mainly degraded by heme oxygenases, it can also be partly exported to the plasma [3]. Moreover, circulating heme can be derived from additional physiological events, such as enucleation of mature erythroblasts, dietary heme absorption and intravascular oxidation of free hemoglobin. Furthermore, pathological conditions can trigger release of heme in the organism, including trauma, hemoglobinopathies, hemorrhages, malaria, bacterial infections, and other diseases, all associated with intravascular hemolysis. Due to free heme toxicity, all these events are characterized by the need to buffer the dangerous properties of heme and to remove it. The binding of heme to Hx allows the transport of heme in the body in a nontoxic form and promotes heme detoxification in the liver through receptor-mediated endocytosis of the complex, followed by heme degradation or reutilization [2,4]. In this way, Hx acts as a pivotal factor in heme detoxification.

Over the years, increasing knowledge has been attained on the critical role of Hx during hemolysis and heme-associated pathologies such as sepsis, sickle cell disease and atherosclerosis [2,3,4,5]. However, heme is not exclusively associated with these kinds of disorders, and emerging evidence supports a critical involvement of heme in cancer [6], thus suggesting a possible implication of Hx also in tumors.

As stated above, exogenous heme can be derived from different sources placed outside the cell. Conversely, endogenous heme is synthetized by all the cells and its level is finely controlled by a plethora of different mechanisms encompassing degradation, distribution among cell compartments, incorporation into hemoproteins and export. Alterations in the levels of both exogenous and endogenous heme can alter cancer cell properties, modifying their metabolism, transcriptional program, proliferation, survival and motility. Moreover, in addition to tumor cell-specific effects, heme can indirectly modulate cancer behavior by shaping the tumor microenvironment [5]. Hx binds heme with an affinity in the picomolar range and, as explained above, it is able to modify its properties, limiting its toxicity and pro-oxidant effects [4]. Moreover, it regulates its distribution in the body, favoring its delivery to the liver. Finally, it can modulate its accumulation inside the cells by promoting its export through the plasma membrane heme exporter FLVCR1a (Feline Leukemia Virus subgroup C Receptor 1a) [6]. For these reasons, Hx could represent a highly interesting player in cancer development.

## 2. Hemopexin as a Cancer Biomarker

The present literature regarding Hx and cancer is restricted. However, Hx has emerged in a series of works looking for possible diagnostic markers for different kinds of tumors.

Analyzing the fucosylated serum proteome from patients with hepatocellular carcinoma (HCC), Mary Ann Comunale and co-workers [7] identified a group of glycoproteins that are altered in HCC patients. Among them, they observed the appearance of fucosylated Hx in the sera of HCC patients when compared to those of normal individuals. In addition, Yiing Lin et al. [8] collected plasma from surgical patients before and after resection of pancreas tumors and examined the proteome expression profiles to search plasma for proteins associated with the disease state. Hx was identified as a biomarker of disease as a plasma protein that correlates with pancreas tumor burden. Along the same line, in a study focused on breast cancer [9], nipple aspirate fluid (NAF) samples from tumor bearing breasts derived from patients with early-stage breast cancer were compared to those obtained from disease-free breasts using quantitative proteomic isotope-coded affinity tag (ICAT) technology and tandem mass spectrometry (MS/MS). The study allowed the observation of a significant over-expression of Hx in tumor-bearing versus disease-free breasts. Similarly, the comparison of sera from non-small cell lung cancer (NSCLC) patients and patients with a benign lung disease (pneumonia), as well as pleural effusion samples from NSCLC patients with those from individuals affected by tuberculosis, showed significant enrichment in Hx levels in the tumor-bearing patients [10], a result confirmed by Wang et al. [11] in a similar study on pleural effusion from lung adenocarcinoma patients. Finally, high levels of Hx were also found in plasma from ovarian cancer patients [12]. Overall, these studies highlighted a possible role for Hx as a biomarker in different kinds of tumors. However, the underlying mechanism accounting for increased Hx levels in body fluids from cancer patients, as well as the effects of circulating Hx on cancer cell properties and disease progression, were far from being elucidated by these studies.

## 3. Hemopexin in Cancer Progression

Recently, additional evidence has pointed to a role for Hx in cancer.

By comparing the pancreatic ductal adenocarcinoma (PDAC) stromal proteome from patient showing lymph-node metastasis (LN+) with that of patients without any metastasis (LN−), it has been observed that Hx expression differs between patients with divergent LN-metastasis status [13]. Particularly, Hx was overexpressed in LN+ PDAC stroma and, thanks to immunohistochemical analyses, it was possible to observe that Hx was mainly expressed in the cancer-cell cytoplasm and stromal fibroblasts. In addition, the authors detected focal expression in lymphocytes and macrophages, whereas no expression was found in vascular endothelium. Beyond these results, the authors observed that Hx expression was also associated with several clinicopathological parameters. As an example, Hx positively associated with venous-invasion and lymphatic-invasion and, although not statistically significant, nerve invasion was higher in patients showing high Hx expression in the tumor stroma. In vitro experiments also demonstrated that Hx enhances the invasive ability of pancreatic cancer cells. Interestingly, tumor size did not differ between the groups, suggesting that Hx is mostly implicated in invasion and metastasis rather than in tumor proliferation. Overall, this study proposed Hx as a previously unrecognized LN-metastasis-associated protein of PDAC and suggested that Hx expression in tumor tissue was potentially associated with LN metastasis and invasion of PDAC.

**Figure 1:**
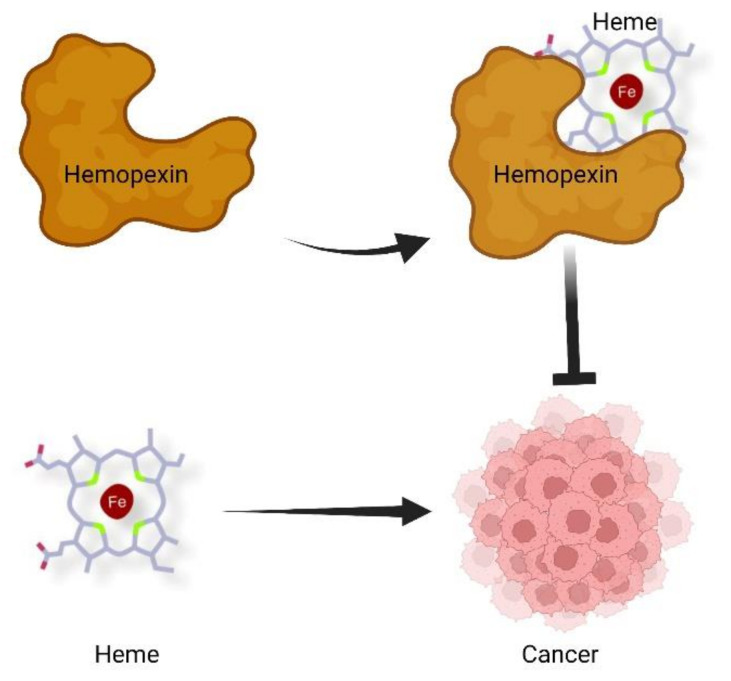
Proposed contribution of heme and Hx to cancer development. According to this model, heme sustains cancer growth and progression, whereas Hx, by scavenging heme, counteracts tumor growth and dissemination. Figure created with BioRender.com, 10 December 2021.

In another study, Canesin et al. [14] analysed the role of heme and Hx in prostate cancer (PCa). By comparing the plasma of patients showing low or high-grade PCa with that from healthy volunteers, the authors detected lower levels of Hx in cancer patients’ samples compared to controls. Similarly, immunohistochemistry analyses showed lower Hx staining in the stroma of tumors than in benign tissues, and low Hx levels in the tumor stroma correlated with poor prognosis and earlier disease relapse. Consistently, PCa tumors established in Hx-null mice were bigger than those raised in Hx proficient controls. Together, these data indicate that, in PCa, Hx expression is crucial to limit tumor growth. In addition to these results, the authors reported that, as in PDAC, Hx expression was higher in metastatic PCa. However, in this case, it is tempting to speculate that Hx up-regulation represents a body attempt to counteract the pro-tumor properties of heme. Indeed, the authors observed that intraperitoneal injection of heme in PCa-bearing mice did not affect tumor volume, whereas it significantly increased local invasion and LN metastasis due to nuclear heme accumulation and to the ensuing transcriptional up-regulation of c-Myc. Similarly, in mice subcutaneously implanted with Lewis lung carcinoma cells heme treatment promoted tumor invasiveness. The effect of heme on c-Myc was ascribed to its ability to intercalate into G-quadruplex (G4) DNA structures, repressor motifs located in the promoter of c-Myc, resulting in their destabilization and, consequently, in the activation of c-Myc transcription. Overall, the study pointed to heme as a promoter of PCa and highlighted a role for Hx as a heme scavenger, counteracting heme-driven cancer progression (Figure 1).

## 4. Conclusions

In conclusion, emerging literature proposes heme and heme-binding proteins as crucial players in different aspects of tumor growth and progression.

Dietary heme has been shown to promote intestinal cancer by different mechanisms, including the production of reactive oxygen species, the eventual formation of carcinogens, as N-nitroso compounds, and the induction of intestinal dysbiosis. Moreover, enhanced endogenous heme biosynthesis, associated with heme export by FLVCR1a, has been reported in different kinds of cancer. This is a strategy adopted by tumor cells to down-modulate the TCA cycle flux and, consequently, the rate of oxidative phosphorylation (OXPHOS), thus limiting the oxidative metabolism [15]. Concomitantly, the increased heme synthesis and heme import observed in cancer [5] can also represent a complementary attempt to improve the efficiency of oxygen utilisation by the OXPHOS machinery in case of oxygen restriction, a frequent condition for tumors. In addition, enhanced heme synthesis and heme accumulation in tumors is also likely intended to support the activity of other specific hemoproteins not related to OXPHOS [5], or to regulate specific transcription factors with known implication in cancer, as the tumor protein P53 (TP53) [16], the BTB Domain and CNC Homolog 1 (BACH1) [17,18,19], and the nuclear factor erythroid 2–related factor 2 (NRF2) [17]. Finally, although it has to be experimentally proven, heme produced by cancer cells can, perhaps, act as a signaling molecule to shape the tumor microenvironment toward a permissive setting [5]. However, the role of heme in cancer, especially in the tumor microenvironment, is still controversial. Indeed, exogenous heme deriving from intratumor hemorrhages could exert an anti-cancer effect rather than a tumor-promoting activity as endogenous heme:M. Costa da Silva and colleagues demonstrated that tumor-associated macrophages (TAMs) exposed to hemolytic red blood cells (RBCs) accumulate iron intracellularly and acquire a M1 pro-inflammatory phenotype, meaning macrophages with anti-tumor properties are able to favor tumor cell death [20]. Similarly, Kayama et al. [21] showed that heme derived from intestinal mucosa bleeding provides intestinal macrophages with a noninflammatory gene expression profile, thus preventing dextran sodium sulfate-induced colitis, a known risk condition for colon cancer. Thus, the impact of heme on cancer, and the precise consequences of endogenous *vs* exogenous heme on the tumor mass and metastasis, show a high level of complexity and elucidation needs further investigation.

Hx is the protein with the highest binding affinity for heme and, as summarized in the paragraphs above, high levels of Hx have been observed in cancer patients’ body fluids, so that it has been proposed as a biomarker for different kinds of cancer, particularly those highly invasive and metastatic (see Table 1 for a comprehensive summary of the observations emerged from the works reported in the present manuscript). The underlying reason for Hx up-regulation in cancer is unknown, but the conclusion emerging from these works is that high Hx levels could support cancer progression. However, the recently published study of Canesin et al. [14] about heme and Hx in PCa indicates that Hx counteracts the pro-tumorigenic effects of heme, likely by its ability to scavenge it. This study provides an alternative explanation for the previous works about Hx as a tumor biomarker: the increased Hx levels in cancer patients body fluids could reflect the attempt of the organism to counteract tumor progression by trying to remove heme. According to this interpretation, Hx is envisioned as a tumor suppressor rather than a tumor-promoting protein. Although this explanation is attractive, as it reconciles most of the current literature on Hx and cancer, some aspects appear still controversial. In vitro experiments in PDAC showed that Hx enhances the invasive ability of pancreatic cancer cells. Moreover, opposite to other kinds of tumor-bearing patients, PCa patients show low Hx levels in their plasma. In addition, enhanced cellular expression of the heme exporter FLVCR1a is a hallmark of cancer, whereas its blockage correlates with decreased tumor cell growth, and Hx has been reported to support FLVCR1a function, thus suggesting a cooperation between the two proteins in the promotion of tumor cell survival/proliferation.

Overall, the role of Hx in cancer appears complex and still controversial. A future, in-depth study of Hx involvement in cancer is desirable and further studies are required to better elucidate the underlying reasons for the apparent discrepancies among the results reported in literature. If additional works confirm a role for Hx as a tumor-promoting protein, its targeting will be viable, as the major body compartments accounting for Hx production are known, as well as its structure. Conversely, if future studies definitively determine a role for Hx as a tumor suppressor, possible therapies will, perhaps, take advantage of already available Hx formulations. Finally, considering the different context-specific and conflicting effects of both heme and Hx on cancer growth and progression, a future definitive classification of Hx as a tumor suppressive/promoting protein could reasonably be excluded, and probably the most promising approach will be to classify and discriminate the precise tumor sub-types that can benefit of each strategy. The study of Hx in the context of cancer appears a promising way to identify future therapeutic actions against cancer.

## Figures and Tables

**Table 1:** Hx as a cancer biomarker.

Tumor	Study Design	Sample	Observations	References
Hepatocellular carcinoma	Patients vs. healthy individuals	Serum	Presence of fucosylated Hx in patients	[7]
Pancreatic tumor	Surgical patients before/after tumor resection	Plasma	Positive correlation between Hx levels and tumor burden	[8]
Breast carcinoma	Tumor bearing breasts vs. disease free breasts	Nipple aspirate fluid	Hx over-expression in tumor-bearing breasts	[9]
Non-small cell lung cancer	Cancer patients vs. pneumonia patients	Serum	Higher Hx levels in cancer patients	[10]
Non-small cell lung cancer	Cancer patients vs. tuberculosis patients	Pleuraleffusion	Higher Hx levels in cancerpatients	[10]
Lung adenocarcinoma	Cancer patients vs. tuberculosis or pneumonia patients	Pleural effusion	Higher Hx levels in cancer patients	[11]
Ovarian cancer	Cancer patients vs. controls	Plasma	Higher Hx levels in ovarian cancer patients	[12]
Pancreatic ductal adenocarcinoma	LN+ PDAC vs. LN- PDAC	Stromal fibroblasts, cancer cell cytosol, lymphocytes, macrophages	Higher Hx levels in LN+ PDAC	[13]
Pancreatic ductal adenocarcinoma	LN+ PDAC	Tumor stroma	Positive correlation between Hx levels and High venous/lymphatic invasion	[13]
Prostate cancer	Patients vs. healthy individuals	Plasma	Lower Hx levels in cancer patients	[14]
Prostate cancer	Tumor vs. benign tissues	Stroma	Lower Hx levels in tumor stroma	[14]
Prostate cancer	Normal tissues vs. tumor-adjacent tissues vs. primary prostate tumors vs. PCa metastasis	Tissues	Higher Hx mRNA levels in metastasis	[14]
